# Equity policies in health plans: accessibility and something more?

**DOI:** 10.11606/s1518-8787.2021055002560

**Published:** 2021-05-19

**Authors:** Bran Barral Buceta, Ramón Bouzas Lorenzo, Andrés Cernadas Ramos, Ángela Fernández da Silva

**Affiliations:** I Universidade de Santiago de Compostela Facultad de Ciencias Políticas y Sociales Departamento de Ciencia Política e Sociología Santiago de CompostelaGalicia España Universidade de Santiago de Compostela. Facultad de Ciencias Políticas y Sociales. Departamento de Ciencia Política e Sociología. Santiago de Compostela, Galicia, España

**Keywords:** Health Systems Plans, Equity in Access to Health Services, Health Equity, Strategies for Universal Health Coverage, Public Nondiscrimination Policies, Qualitative Research

## Abstract

**OBJECTIVE:**

To examine the approach adopted by the health plans of the autonomous communities of Spain, verifying the weight given to the concept of equity; to detect referenced communities or situations, as well as to distinguish the perspective of approaching it, from access, equity or equalization.

**METHODS:**

Qualitative study, of content analysis using Nvivo12, carried out in 2020 on health plans in force since 2019 in the different regions (autonomous communities) of Spain. Sixteen current regional health plans were compiled to establish base categories (equity, accessibility and equality) and determine associated terms using Nvivo12, from which a content analysis was performed.

**RESULTS:**

The concept of equity is not emphasized in the regional health plans and its relevance is surpassed by the concepts of accessibility and equality. The use of these three concepts is associated with various categories indicating circumstances, conditions or groups to which the plans give greater attention.

**CONCLUSIONS:**

The results obtained coincide with previous studies on the contents and orientation of health plans, revealing a discrete presence of the concept of equity in the approaches adopted, although this does not undermine the alignment of health policies with the visions emanating from transnational organizations. It is detected the existence of a group to which special attention is given from the accessibility approach, the population with functional diversity.

## INTRODUCTION

In recent decades, the conception of health and its determinants has adopted more proactive and preventive approaches in line with principles, including the defense of equity, emphasized by the World Health Organization (WHO)^1^^–^^4^.

Interpretations of the concept of equity are broad^5^^,^^6^ and include the alignment with state anti-interventionism, the defense of equality of basic capabilities and maximum state support, and the defense of equity in access^6^. The first positions are far from the main debates on equity, since they are based on the premise that inequality is inherent to freedom of choice, although some authors opt for establishing a set of basic services^7^^,^^8^. The other alternatives, to a greater or lesser extent, favor the design of policies aimed at attenuating or eradicating health inequities. Among the latter, states play a more relevant role^9^, with proposals aimed mainly at defending universality –the Beveridge Report^10^ being a reference point–, providing social security systems and introducing the means-testing programs^11^^,^^12^ –a sort of social insertion income or positive discrimination–, all of them forms of intervention that are not free of debate^13^^–^^15^.

The WHO^(^^1^^–^^4^^)^ approached the concept of “basic capabilities” based on the contributions of Sen^16^^,^^17^ and Nussbaum^18^^,^^19^, accepting personal responsibilities in the state of health and defending action on any factor causing inequity –unfavorable socioeconomic, socio-demographic, geographic, ethnic or gender conditions– that may introduce inequalities in well-being. The concept of “avoidable inequality”^5^ developed by the WHO derives from this proposal.

Spain, like other European and Organization for Economic Cooperation and Development (OECD) countries, included the principle of equity in health policies, emphasizing its assimilation during the first decade of this century and reviewing its survival in the context of the 2008 financial crisis^20^^–^^23^.

Setting aside the treatment of the relevance of the different applications of equity to social reality, this paper is interested in the way in which the health services of the Autonomous Communitiesᵃ (AC) introduce the issue of equity and which groups or circumstances they consider when addressing it. The objective is to reveal the presence of the concept of equity in health plans, to identify the main associated topics and the treatment given to them, contrasting their orientations in the framework of public health policies with the visions sponsored by the Ministry of Health, the European Union (EU) and the WHO.

## METHODS

### Design

Following the guidelines of constant comparative analysis^24^, by examining the contents^25^^–^^27^ supported by a qualitative research support application –Nvivo12–, the health plans of the different AC were reviewed.

### Sample

The study includes the general health plans in force –newly elaborated or extended– promoted by 16 AC.

- Andalusia: IV Andalusian Health Plan (2013–2020)
- Aragon: Aragon Health Plan 2030
- Balearic Islands: Strategic Plan 2016–2020
- Canary Islands: Canary Islands Health Plan 2016–2017: Between the crisis and the transformation for innovation in the management of health services
- Cantabria: Cantabria Health Plan 2014–2019
- Castile-La Mancha: 20-20 Health and Social Welfare Strategy: The Castile-La Mancha model
- Castile and León: IV Health Plan of Castile and León (2020)
- Catalonia: Catalan Health Plan 2016–2020
- Valencian Community: IV Health Plan 2016–2020 Valencian Community
- Extremadura: Extremadura Health Plan 2013–2020
- Galicia: Sergas Strategy 2020
- La Rioja: III Health Plan of La Rioja (2013–2020)
- Navarre: Navarre Health Plan 2014–2020
- Basque Country: Euskadi Health Plan 2013–2020
- Principality of Asturias: Social Health Plan of the Principality of Asturias (2018–2021)
- Region of Murcia: Health Plan 2010–2015 of the Region of Murcia

These plans are part of the study because they meet the criteria of homogeneity, representativeness, relevance and completeness proposed by Bardin^25^. The aim was to analyze the most current general health plans possible. For this reason, it was not possible to analyze any plan of the autonomous region of Madrid since, following the rule of homogeneity, it was not possible to find a plan with these characteristics, as all the plans collected were partial and specific.

### Analysis

The literature identifies factors or determinants of health that, due to the influence of transnational reference institutions, were expected to be reproduced, to a greater or lesser extent, in health plans.

However, content analysis from Bardin's^25^ perspective, especially the emergent approach^24^ adopted, forces us to extract categorizations and meanings from the object of study, and not to predefine them.

Thus, during the pre-analysis phase^25^, we proceeded to select the fragments of the plans that addressed contents related to equity. From this first approach, several key items were detected, and the selection of text fragments was expanded –in consequence of the emerging perspective adopted– to two other fields of meanings that are synthesized in the concepts of “Accessibility” and “Equality,” which have been called nodes or base categories (BC). The concept “node” is specific to the terminology of the Nvivo application and refers to a category that groups similar signifiers: each category operates as a set of terms related to the one that names it^b^.

Subsequently, content analysis was carried out and, as is typical of a constant comparison approach, new subcategories^c^ were formed according to the frequency of occurrence. Some of them are widely reported in the literature consulted, and others, less common, are derived from the design adopted. As a result, the following key categories (KC) were established: Quality, Functional Diversity, Gender, Vulnerable Social Groups, Immigration, Older Adults, Rural and Mental Illness.

The difference between BC and KC lies in their function in the analysis process: the “base” articulate the selection of the analyzed plan segments (they operate as framework signifiers); and the “key” determine the content within that selection. In other words, the base categories are the containers (the text segments included in the analysis) of what is being discussed (key categories). This is another of the reasons that led the research team to accept “multicoding” in these three cases, since it allows a thematic analysis of the KC adjusted to each of the BC and, on this basis, to establish the similarities and differences between the different paradigms represented.

Chart 1 shows the operationalizations made to form the various base and key categories mentioned in the article, as well as the terms or lexemes included in the analysis^d^.

**Chart:** List of categories and terms or lexemes applied and included in the coding.

Category		Terms or lexemes used
Base Categories	Equity	Equity, Equitable*, Inequitable*
	Equality	Equality, Inequality*, Egalitarian*
	Accessibility	Accessibility, Accessible, Access, Inaccessible, Inaccessibility
Key Categories	Functional diversity	Functional diversity, Disability*, Disability, Dependent*, Dependency, Independence
	Rural	Rural, Periphery, Small/remote population centers, Dispersed, Dispersion, Difficult to access, Distant*, Zone*, Geography*, Geographical*
	Vulnerable social groups	Social group, Social groups, Groups at risk, Vulnerability, Vulnerabl*, Disadvantaged*, Collective*
	Gender	Gender, Man*, Woman*, Violence, Abuse, Sexism
	Immigration	Immigration, Immigrant, Race, Ethnicity, Ethnic*, Language, Speech, Foreign*.
	Older Adults	Older Adults*, Older Adults/Third age, age, aging, ancient*
	Quality	Quality
	Mental	Mental*

* We selected words that begin in this way, so that they can be used to avoid gender or plurals. This is a practice specific to the Nvivo program and its search criteria, expressed in this way. These terms were reviewed case by case to observe their relevance, discarding those that did not have a meaning consistent with what was searched for. All the lexemes presented in the table were searched in their corresponding Spanish translation, since this is the original language of most of the plans (also in Catalan), and the table shows the lexemes or words in english for a better understanding of the methodology applied.

## RESULTS

**Figure 1:**
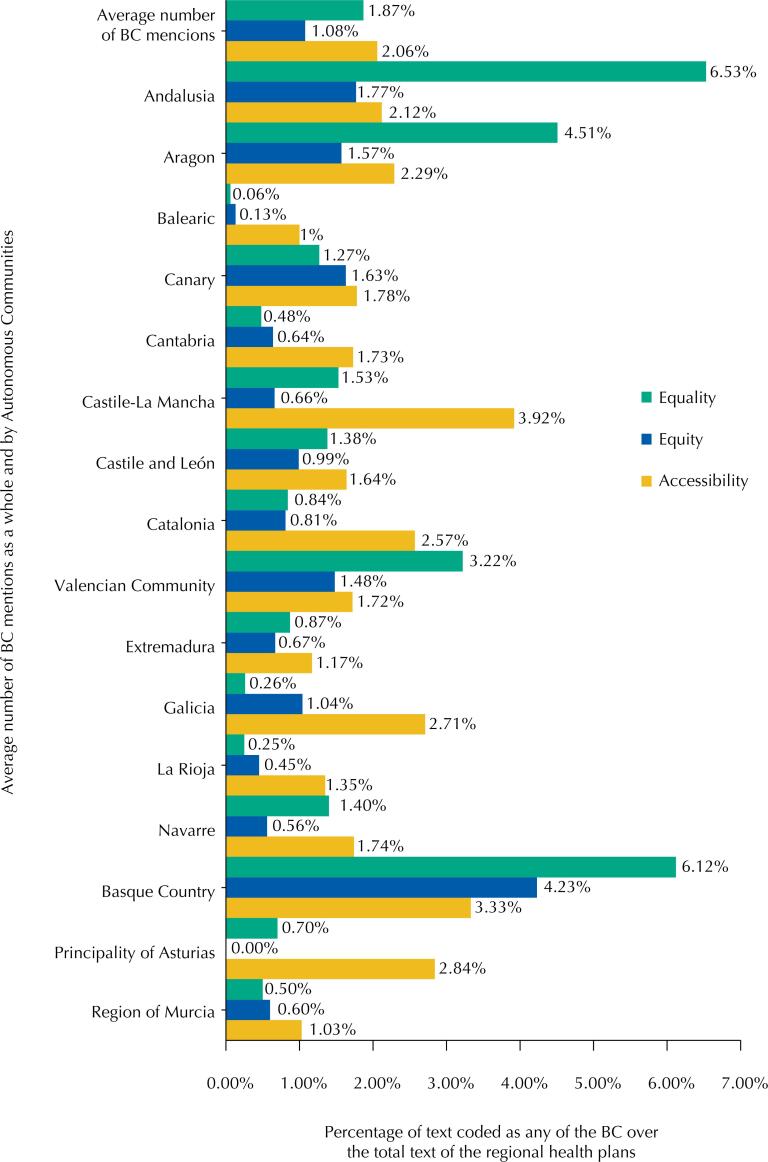
Percentage of text coded according to the base categories over the total text included in the autonomous plans.

First, the extent to which health plans contain references to the concepts associated with health equity was determined (Figure 1).

The most widely detected concept in the health plans is Accessibility, 2.06% (on average), higher than the treatment of the remaining BC in 11 of the regional health plans analyzed. However, the concept of Equality reaches higher levels of mention (in terms of maximums, with a lower average of 1.87%), standing out in 6.53% of the contents of the Andalusia health plan, 6.12% of the Basque Country plan and 4.51% in the case of Aragon.

Equity concept is more relevant in the health plan of the Basque Country (4.23% of contents) and, to a lesser extent, in the Andalusian autonomous plan (1.77%) and in that of the Canary Islands (1.67%), obtaining an average coding of 1.08%, the lowest of the three BC.

The concept of accessibility reached its highest level in the regional plan of Castile-La Mancha (3.92%), followed by that of the Basque Country (3.33%) and Asturias (2.84%).

The lack of content found in some plans is also significant: the treatment of the Equality concept is scarce –less than 0.25%– in the plans of Asturias, the Balearic Islands and La Rioja; the Equity concept is minimally addressed –less than 0.7%– in the plans of the Balearic Islands, Cantabria and Castile-La Mancha; and the term Accessibility reaches its lowest figures –less than 1.2%– in the plans of the Balearic Islands, Murcia and Extremadura.

But what does it mean when referring to equity, equality and accessibility?

Although, as mentioned above, the issues associated with BC – Equity, Equality and Accessibility – are incorporated in practice in all the health plans, the treatment of KC differs significantly, as shown by the distribution of mentions in the regional health plans (Table 1).

**Table 1:** Mentions of base and key categories in the regional health plans by frequency of coding (out of total coded by category).

	Accessibility	Quality	Functional Diversity	Equity	Gender	Vunerable Social Groups	Equality	Immigration	Older Adults	Mental Illness	Rural	Avarage number of mentions over Total per CA
Andalucia	5.38%	10.86%	4.34%	8.01%	7.61%	5%	16.90%	5.36%	0.67%	6.52%	10.10%	7.34%
Aragon	3.27%	1.33%	0.91%	4.14%	0.74%	3.50%	7.99%	5.85%	4.67%	2.10%	8.75%	3.93%
Balearic	2.16%	0.27%	0.16%	0.54%	0%	0%	0.17%	1.17%	0%	0%	0%	0.41%
Canary	6.13%	6.63%	0.99%	10.14%	0.34%	3.88%	5.24%	1.17%	0%	2.05%	3.52%	3.64%
Cantabria	16.56%	3.71%	18.85%	11.04%	3.69%	8.13%	5.52%	10.62%	5.71%	52.70%	5.44%	12.91%
Castile-La Mancha	2.78%	0.80%	3.28%	0.81%	0.11%	0.48%	1.25%	0.39%	0.67%	0%	3.47%	1.28%
Castile and León	6.93%	2.12%	7.18%	7.51%	2.61%	1.67%	7.10%	4.42%	0.46%	6.52%	9.74%	5.11%
Catalonia	5.99%	42.71%	1.53%	3.68%	58.55%	19.82%	2.65%	0%	59.18%	2.09%	0%	17.84%
Valencian Community	8.41%	3.98%	4.13%	12.43%	3.98%	21.28%	17.63%	16.17%	0.64%	0%	0%	8.06%
Extremadura	10.98%	7.43%	13.31%	11.92%	10.34%	5.88%	10.23%	9.97%	17.08%	2.17%	47.28%	13.33%
Galicia	1.61%	1.06%	0%	1.17%	0%	0%	0.18%	0%	0%	0%	0%	0.37%
La Rioja	10.97%	5.04%	22.17%	6.91%	6.59%	7.55%	2.55%	30.04%	6.26%	8.70%	6.01%	10.25%
Navarre	6.42%	3.45%	9.32%	3.86%	0.68%	7.27%	6.08%	7.60%	0.96%	2.11%	3.11%	4.62%
Basque Country	6.39%	8.22%	1.94%	14.58%	4.03%	7.65%	13.86%	0.81%	2.03%	1.98%	0.62%	5.65%
Principality of Asturias	3.02%	1.59%	6.86%	0%	0.45%	4.37%	0.91%	0.39%	1.67%	10.87%	1.29%	2.86%
Region of Murcia	3%	0.80%	5.03%	3.26%	0.28%	3.52%	1.74%	6.04%	0%	2.19%	0.67%	2.41%
Total category mentions	100.00%	100.00%	100.00%	100.00%	100.00%	100.00%	100.00%	100.00%	100.00%	100.00%	100.00%	100.00%

Note: The colors are based on the intensity and direction of the coding; those closer to red denote a lower percentage of mentions; the greens are higher; and the yellow tones are more moderate. In this case, it represents the values in columns and therefore corresponds to the percentage of text segments that have been coded in each category according to the individual total of each one of them. As a result, it shows which communities emphasize in greater or lesser extent each theme.

In the columns that mention the number of pages of the plans, an indicator is created in the last column that shows the ratio of average coding per page of the plan, which allows relating the percentage of coding of each community with its number of pages: those that are greener indicate a higher proportion between pages and coding, and therefore that greater attention is given to equity; those closer to red show communities that give less attention.

From the point of view of the thematic diversity covered, the least variety of KC is detected in the plans of Galicia and the Balearic Islands – Galicia only deals with the concept of Quality and the Balearic Islands with Immigration and Quality – while several autonomous health plans (Andalusia, Aragon, Cantabria, Castile and Leon, Extremadura, La Rioja and Navarra) cover all the KC, albeit with unequal attention.

With respect to the intensity of thematic treatment, the plan for Catalonia stands out, with more than half of the mentions of three KC in the regional plans: Older Adults (59.18%), Gender (58.55%) and Quality (42.71%). Two other regional plans have the most references to two KC: Mental Illness (52.7%), in the case of Cantabria, and Rural (47.28%), in Extremadura.

With less intensity, others KC in which health plans stand out are: Immigration (La Rioja, 30.04%), Vulnerable Social Groups (Catalonia, 21.28%; Valencia, 19.82%) and Functional Diversity (La Rioja, 22.17%; Cantabria, 18.85%).

On the other hand, it is important to place the treatment of the topics in the context of their relevance in the plans, since the fact that a plan accumulates the greatest number of mentions of a category does not necessarily imply that it has greater importance than the others, as will be seen below.

Firstly, it is important to note that the BC have a greater presence, a logical fact given the text selection in function of them. In this regard, the BC with the highest average number of codification is Accessibility, with 36.09% of the mentions, followed by Equality, 22.77%, and Equity, 16.24%. The latter maintains a percentage of mentions similar to that achieved by the KC Functional Diversity (13.72%), one of the most important themes in the selected fragments.

The remaining KC are subsumed in terms of number of mentions: Vulnerable Social Groups (3.77%), Older Adults (2.42%), Immigration (2.19%), Gender (1.17%), Rural (1.31%), Quality (0.30%) and Mental Illness (0.03%).

Evaluating the importance of the KC outside of the BC facilitates a more refined thematic analysis by community (Table 2). Thus, the Functional Diversity category stands out above the others (48.36% on average, with Aragon, Catalonia and Galicia below 30% of mentions). It is necessary to go down to 16.56% of the average codifications to find the following KC, Vulnerable Social Groups – mentions of this issue in the cases of the Valencian Community, the Basque Country, the Canary Islands, Catalonia and Aragon, all of which account for more than 20% of the thematic coding carried out by the AC. The third category, Immigration, with 11.40%, stands out for its importance in the Balearic Islands with 55.37% and to a lesser extent Aragon and the Valencian Community with numbers around 20%.

**Table 2:** Percentage of coding of the issues addressed by the KC (on total coded by autonomous health plans).

	Quality	Functional Diversity	Gender	Vunerable Social Groups	Immigration	Older Adults	Mental Illness	Rural	Total
Andalucia	2.62%	47.76%	8.55%	16.54%	10.48%	1.45%	0.21%	12.41%	100.00%
Aragon	0.58%	18.08%	1.48%	20.98%	20.72%	18.53%	0.13%	19.50%	100.00%
Balearic	1.47%	43.16%	0.00%	0.00%	55.37%	0.00%	0.00%	0.00%	100.00%
Canary	4.88%	33.77%	1.19%	39.58%	7.12%	0.00%	0.13%	13.32%	100.00%
Cantabria	0.30%	73.85%	1.48%	9.57%	7.41%	4.45%	0.54%	2.39%	100.00%
Castile-La Mancha	0.43%	81.14%	0.30%	3.58%	1.71%	3.28%	0.00%	9.56%	100.00%
Castile and León	0.48%	71.93%	2.69%	5.04%	7.87%	0.91%	0.19%	10.89%	100.00%
Catalonia	3.55%	5.83%	22.77%	22.77%	0.00%	45.07%	0.02%	0.00%	100.00%
Valencian Community	0.64%	29.42%	2.89%	45.61%	20.52%	0.92%	0.00%	0.00%	100.00%
Extremadura	0.60%	49.75%	3.94%	6.60%	6.63%	12.73%	0.03%	19.71%	100.00%
Galicia	100.00%	0.00%	0.00%	0.00%	0.00%	0.00%	0.00%	0.00%	100.00%
La Rioja	0.34%	68.18%	2.07%	7.00%	16.45%	3.83%	0.06%	2.07%	100.00%
Navarre	0.55%	68.81%	0.52%	16.15%	9.99%	1.39%	0.03%	2.56%	100.00%
Basque Country	3.26%	35.43%	7.58%	42.19%	2.68%	7.46%	0.12%	1.28%	100.00%
Principality of Asturias	0.39%	77.62%	0.53%	14.93%	0.78%	3.79%	0.33%	1.63%	100.00%
Region of Murcia	0.23%	69.01%	0.41%	14.51%	14.74%	0.00%	0.09%	1.02%	100.00%
Average number of BC mentions per AC	7.52%	48.36%	3.52%	16.56%	11.40%	6.49%	0.12%	6.02%	100.00%

Note: For greater clarity about the content importance within each plan, the relative frequency of mention of each of the KC is detailed and the specific value of each of them is analyzed in relation to the others (excluding, as mentioned above, the BC). This highlights those topics or issues addressed to a greater or lesser extent by each community. The use of colors follows the previously applied pattern, but in this case responds to the numbers located in the lines, thus showing the relative importance of the KC in each of the plans individually and, therefore, in each community.

The supremacy of the concept of Functional Diversity is present in most of the AC, being one of the most prominent targets. It is especially visible in the plans of Castile-La Mancha, Asturias, Cantabria, Castile and Leon, Region of Murcia, La Rioja and Navarra. Galicia, which deals exclusively with equity in health in relation to quality of care, is not included.

The centrality of Functional Diversity associated with the Accessibility category can be explained by the measures set out in the health plans and how their causes are argued: from access to the healthcare infrastructures to the adaptation of web pages to more accessible formats for different types of users, including the establishment of home care that facilitates the provision of healthcare under equal conditions.

The categories Functional Diversity, Vulnerable Social Groups and Immigration focus attention on the majority of contents that express the need to promote equity in health, articulating policies or measures that seek to improve the situation in which are these groups.

Thus, since the semantic fields of each category imply not only the consideration of a condition or circumstances, but also allude to the portion of the population affected by them, the health actions aimed at improving the situation of other groups are also the main ones among the plans examined: Catalonia with respect to the Older Adults (45.07%) and Gender (22.77%); or Extremadura and Aragon, when dealing with Rural KC (19.71% and 19.50%, respectively).

**Figure 2:**
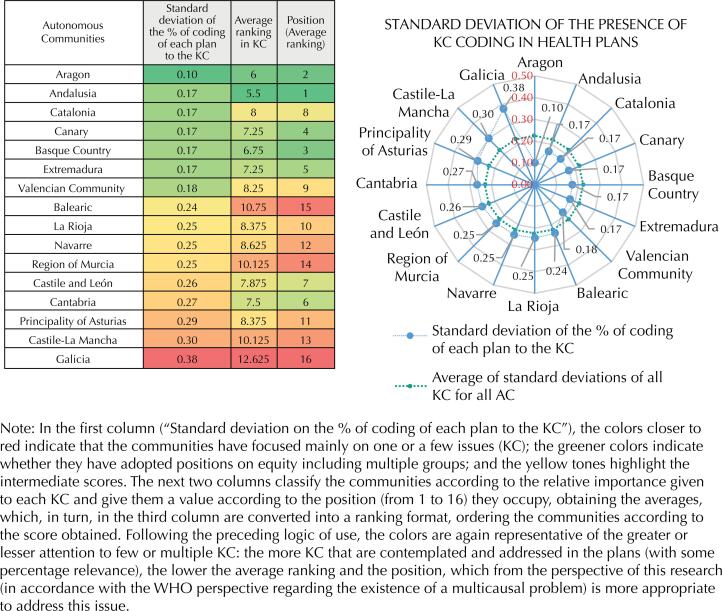
Ranking of the AC according to the attention they give to the different AC in their plans.

Based on all the above data, the situation of each health plan has been represented (Figure 2) according to the relevance given to each KC.

This figure shows that there are several AC whose approach to equity is concentrated in few KC, while others address more targets and, therefore, understand that it is a broader issue. The first group includes the Balearic Islands, La Rioja, Navarra, Region of Murcia, Castile and León, Cantabria, Asturias, Castile-La Mancha and, finally, Galicia. The second group includes Aragón, Andalusia, the Canary Islands, Catalonia, Extremadura, Basque Country and Valencian Community, all of them below the average standard deviation and, therefore, with a tendency to observe and consider more groups than the others.

## DISCUSSION

Knowing what is the approach of each community to equity, on which groups they are focusing their health policies and with what orientation, implies checking which issues are not being addressed, raising the question about this absence considering the perception that transnational actors such as the WHO and the EU have of the theory of basic capabilities proposed by Sen^3^^,^^4^^,^^16^^,^^17^^,^^21^.

This qualitative study corroborates the findings of previous research that autonomous health plans contain few explicit references to the concept of equity in health^20^^–^^23^^,^^28^. The plans focus their attention on addressing specific diseases, the incorporation of health technologies and the reorganization of services, leaving the issue of equity in the second place, which is only revealed, indirectly, by the demand for improved access to health services, adopting, in short, a vision oriented to the perspective of equal access and not in line with the proposal of Sen and the WHO on the equality of capabilities and attention to social determinants.

Furthermore, this access is not considered by all the AC as a general and multicausal factor with various groups or key issues to be considered, but rather specific groups are identified, among which people with Functional Diversity, the main target at which health equity policies are aimed, stands out. In second place are references to other important groups such as Vulnerable Social Groups and Immigrants. This does not prevent us from highlighting that the analysis confirms that health plans link the category of Vulnerable Social Groups to situations of economic discrimination, a relationship evidenced by transnational organizations such as the WHO as a source of inequity associated with poverty and health worsening^5^.

Attention to issues such as gender, quality, older adults, rural areas or mental illness is not uniformly addressed in the regional plans. However, this fact does not make them irrelevant; on the contrary, their absence is evidence of the lack of a multidimensional perspective on health inequities. All of this has serious repercussions, as it can lead to a lack of care for these groups, who have additional difficulties in becoming full users of the health service^20^^–^^23^^,^^28^^,^^29^.

From the point of view of the promoter of the health plans –the Autonomous Administrations– on the one hand, we can distinguish communities that approach equity as a very focused issue on one or two groups and those that deal with the problem in a more comprehensive, global way, closer to the concept of basic capabilities, although none of them go beyond the accessibility factor and do not fully consider the other components of health inequity as defended by the WHO and the literature on the Spanish case^20^^–^^23^^,^^28^^–^^30^.

Although the conception of health policy design may suffer from insufficient specific attention to certain groups, implementation should consider them and thus avoid serious inequities in the levels of service use: access to the same service does not guarantee equal care if, for example, some users have no financial resources to pay for their medication, have difficulties in understanding the language used to attend them or are unable to visualize a prescription. The quality of their care, even the care itself, is at risk^6^^,^^16^^,^^17^^,^^19^.

All the key categories detected are perfectly applicable to all the AC. Not highlighting them does not result from the lack of specific groups, but from a political choice of the target of the plans.

Confirming previous studies that indicated the same direction^20^^–^^23^^,^^28^^–^^30^, the results certify the lack of a greater reflection of equity in the plans and the general failure to address the key issues. Relying on implementation alone to cover the shortcomings of planning and, as in this case, to mitigate a meager treatment of equity, is not a satisfactory practice.

Implementation is an area beyond the scope of this paper. It is only in the study of the implementation of what the health plans propose that it will be really possible to verify how and in what way the starting ideas^20^^,^^31^ that have been the object of this analysis are shown. Thus, hopefully, this research will stimulate further studies on the subject and will serve as a framework for more in-depth analysis of current and future health plans.

## Footnotes

a According to the current Constitution of 1978, Spain is a State territorially decentralized into 17 communities with broad powers over various matters and services, including health. The central Administration reserves for itself the competencies on external health and the coordination of the health system in general, transferring the other competencies on health services to the AC. Given the current high level of territorial decentralization, there is not a single health system in the country, but rather 17 subsystems, with some communities offering different services, although in basic aspects the system is relatively homogeneous for the whole of the State.

b These three categories contradict the “mutual exclusion” criterion, basic to the configuration of categories, something that the research team sought to avoid. This circumstance is one of the exceptions that Bardin^25^ contemplates for the phenomenon he calls “multicoding.” Given the imprecision with which sometimes reference is made to ideas or items conceptualized differently in the literature, these elements can be coded without necessarily excluding them from belonging to another category. This is the case with the BC, which are sometimes used as synonyms. In order to establish whether there are issues specific to each of them and, in turn, to find those that are transversal to all three, we decided to use this option, so that in cases where text segments that mention 2 or even 3 BC, the issues addressed are considered to be part of the three categories.

c These subcategories respond to the usual configuration of the categories, being completely exclusive and being contained in some or all of the above.

d A result of the analysis of the frequency of lexemes in the content of the plans and thanks to the Nvivo program tool that allows this search criterion by lexeme, also supported by the extension using the main dictionaries of synonyms and the native linguistic competence of the research team.

## References

[1] 1. World Health Organization. Health21: the health for all policy framework for the WHO European Region. Copenhagen (DK): WHO Regional Office for Europe; 1999 [cited 2019 Sep 23]. (European Health for All Series; n° 6. Available from: http://www.euro.who.int/__data/assets/pdf_file/0010/98398/wa540ga199heeng.pdf

[2] 2. Marmot M; Commission on Social Determinants of Health. Achieving health equity: from root causes to fair outcomes. Lancet. 2007;370(9593):1153-63. https://doi.org/10.1016/S0140-6736(07)61385-3 10.1016/S0140-6736(07)61385-3 17905168

[3] 3. Dahlgren G, Whitehead M. Policies and strategies to promote social equity in health; background document to WHO – Strategy paper for Europe. Stockholm (SE): Institute for Future Studies; 1991 [cited 2019 Sep 24]. Available from: https://core.ac.uk/download/pdf/6472456.pdf

[4] 4. World Health Organization. Working towards achieving the Sustainable Development Goals: a WHO toolkit. Geneva (CH): WHO; 2018 1991 [cited 2019 Sep 24]. Available from: https://apps.who.int/iris/handle/10665/274261

[5] 5. Whitehead M. The concepts and principles of equity and health. Int J Health Serv.1992;22(3):429-45. https://doi.org/10.2190/986L-LHQ6-2VTE-YRRN 10.2190/986L-LHQ6-2VTE-YRRN 1644507

[6] 6. Cernadas Ramos A. La salud y el acceso a los sistemas sanitarios públicos: desigualdades e inequidades. Madrid: Editorial Síntesis; 2010.

[7] 7. Enthoven AC. Is consumer choice and competition in health care the wave of the future? Hosp Financ Manage. 1980;34(11):12-5, 18, 20 passim. 10249032

[8] 8. Nozick R. Anarquía, Estado y utopía. [S.l.]: Editorial Innisfree; 2014.

[9] 9. Navarro V, Quiroga A. Políticas de estado de bienestar para la equidad. Gac Sanit. 2004;18(4):147-57. 10.1157/13062264 15171872

[10] 10. Beveridge WH. Social insurance and allied services: report by Sir William Beveridge. Bull World Health Organ. 2000;78(6):847-55. 2000 [cited 2019 Sep 27];78(6):847-55. Available from: https://apps.who.int/iris/handle/10665/57560 PMC2560775 10916922

[11] 11. Korpi W, Palme J. The paradox of redistribution and strategies of equality: welfare state institutions, inequality, and poverty in the western countries. Am Sociol Rev. 1998;63(5):661-87. https://doi.org/10.2307/2657333

[12] 12. Diderichsen F. Income maintenance policies: determining their potential impact on socioeconomic inequalities in health. In: Mackenbach J, Bakker M, editors. Reducing inequalities in health: a European perspective. London (UK): Routledge; 2002. p. 53-66.

[13] 13. Sojo A. Reformas de gestión en salud en América Latina: los cuasimercados de Colombia, Argentina, Chile y Costa Rica. Santiago de Chile: Editorial Cepal; 2000.

[14] 14. Altman D, Frist WH. Medicare and Medicaid at 50 years: perspectives of beneficiaries, health care professionals and institutions, and policy makers. JAMA. 2015;314(4):384-95. https://doi.org/10.1001/jama.2015.7811 10.1001/jama.2015.7811 26219056

[15] 15. Rice T, Unruh LY, Ginneken E, Rosenau P, Barnes AJ. Universal coverage reforms in the USA: fom Obamacare through Trump. Health Policy. 2018;122(7):698-702. https://doi.org/10.1016/j.healthpol.2018.05.007 10.1016/j.healthpol.2018.05.007 29804633

[16] 16. Sen A. Equality of What? In: McMurrin S, editor. Tanner lectures on human values. Vol. 1. Cambridge (UK): Cambridge University Press; 1980.

[17] 17. Sen A. Inequality reexamined. Oxford (UK): Clarendon Press; 1992.

[18] 18. Nussbaum MC. Frontiers of justice: disability, nationality, species membership. Cambridge, MA: Harvard University Press; 2009.

[19] 19. Nussbaum MC. Creating capabilities: the human development approach. Cambridge, MA: Harvard University Press; 2011.

[20] 20. Comisión para Reducir las Desigualdades Sociales en Salud en España. Propuesta de políticas e intervenciones para reducir las desigualdades sociales en salud en España. Gac Sanit. 2012;26(2):182-9. https://doi.org/10.1016/j.gaceta.2011.07.024 10.1016/j.gaceta.2011.07.024 22112713

[21] 21. Marmot M, Allen J, Bell R, Bloomer E, Goldblatt P; Consortium for the European Review of Social Determinants of Health and the Health Divide. WHO European review of social determinants of health and the health divide. Lancet. 2012;380(9846):1011-29. https://doi.org/10.1016/S0140-6736(12)61228-8 10.1016/S0140-6736(12)61228-8 22964159

[22] 22. Puyol A. Ética, equidad y determinantes sociales de la salud. Gac Sanit. 2012;26(2):178-81. https://doi.org/10.1016/j.gaceta.2011.08.007 10.1016/j.gaceta.2011.08.007 22115543

[23] 23. Urbanos-Garrido R. La desigualdad en el acceso a las prestaciones sanitarias. Propuestas para lograr la equidad. Gac Sanit. 2016;30 Supl 1:25-30. https://doi.org/10.1016/j.gaceta.2016.01.012 10.1016/j.gaceta.2016.01.012 27004770

[24] 24. Glaser BG, Strauss AL. The discovery of grounded theory: strategies for qualitative research. New York: Aldine de Gruyter; 1967.

[25] 25. Bardin L. Análisis de contenido. 2. ed. Madrid: Ediciones Akal; 1996.

[26] 26. Mayring P. Qualitative content analysis. Forum Qual Soc Res. 2000 [cited 2019 Nov 17];1(2):20. Available from: http://www.qualitative-research.net/index.php/fqs/article/view/1089/2385

[27] 27. Silva SAG, Duarte RG, Castro JM. Transfer of knowledge in international cooperation: the Farmanguinhos-SMM case. Rev Saude Publica. 2017;51:103. https://doi.org/10.11606/s1518-8787.2017051006249 10.11606/S1518-8787.2017051006249 PMC5697924 29166441

[28] 28. Whitehead M, Dahlgren G. Levelling up (part 1): a discussion paper on concepts and principles for tackling social inequities in health. Copenhagen (DK): WHO Regional Office for Europe; 2006 [cited 2019 Dec 14]. (Studies on social and economic determinants of population health; n°.2). Available from: https://apps.who.int/iris/handle/10665/107790

[29] 29. Borrell C, Peiró R, Ramón N, Pasarín MI, Colomer C, Zafra E, et al. Desigualdades socioeconómicas y planes de salud en las comunidades autónomas del Estado español. Gac Sanit. 2005;19(4):277-85. https://doi.org/10.1157/13078025 10.1157/13078025 16050962

[30] 30. Briones-Vozmediano E, Vives-Cases C, Peiró-Pérez R. Gender sensitivity in national health plans in Latin America and the European Union. Health Policy. 2012;106(1):88-96. https://doi.org/10.1016/j.healthpol.2012.03.001 10.1016/j.healthpol.2012.03.001 22465154

[31] 31. Fiuza Pérez MD, Aguiar Rodríguez JF, Monzón Batista N. Una década de reflexión sobre los planes de salud en España. Informe SESPAS 2010. Gac Sanit. 2010;24 Supl 1:37-41. https://doi.org/10.1016/j.gaceta.2010.10.005 10.1016/j.gaceta.2010.10.005 21074905

