# Supplementary material for: Equity policies in health plans: accessibility and something more?
Source: Rev Saude Publica. 2021 May 19;55:31. doi: 10.11606/s1518-8787.2021055002560 (PMC8139844; doi:10.11606/s1518-8787.2021055002560)
Supplement: Translation

# Translation: Políticas de equidad en los planes de salud: accesibilidad ¿y algo más?

**Authors:** Bran Barral Buceta, Ramón Bouzas Lorenzo, Andrés Cernadas Ramos, Ángela Fernández da Silva

**Affiliations:** I Santiago de CompostelaGalicia España Universidade de Santiago de Compostela. Facultad de Ciencias Políticas y Sociales. Departamento de Ciencia Política y Sociología. Santiago de Compostela, Galicia, España

**Keywords:** Planes de Sistemas de Salud, Equidad en el Acceso a los Servicios de Salud, Equidad en Salud, Estrategias para Cobertura Universal de Salud, Políticas Públicas de no Discriminación, Investigación Cualitativa

## Abstract

**OBJETIVO:**

Examinar el enfoque adoptado por los planes de salud de las comunidades autónomas de España verificando el peso otorgado al concepto de equidad; detectar a qué colectivos o situaciones se hace referencia; así como distinguir la perspectiva de abordaje del mismo, desde el acceso, la igualación o la equiparación.

**MÉTODOS:**

Estudio cualitativo, de análisis de contenido mediante Nvivo12, realizado en 2020 sobre planes de salud en vigencia a fecha de 2019 en las diferentes regiones (comunidades autónomas) de España. Se han recopilado 16 planes de salud regionales vigentes para establecer categorías base (equidad, accesibilidad e igualdad) y determinar términos asociados a través de Nvivo12, a partir de los que se realizó un análisis de contenido.

**RESULTADOS:**

El concepto de equidad no resulta destacado por los planes de salud autonómicos y su relevancia es superada por los conceptos de accesibilidad e igualdad. El empleo de estos tres conceptos está asociado a diversas categorías que connotan circunstancias, condiciones o colectivos a los que los planes prestan mayor atención.

**CONCLUSIONES:**

Los resultados obtenidos coinciden con estudios previos sobre los contenidos y orientación de los planes de salud, revelando una presencia discreta del concepto de equidad en los enfoques adoptados, sin que ello menoscabe el alineamiento de las políticas de salud respecto de las visiones emanadas de organizaciones transnacionales. Se detecta la existencia de un colectivo al que se presta especial atención desde el enfoque de accesibilidad, la población con diversidad funcional.

## INTRODUCCIÓN

En las últimas décadas, la concepción de la salud y sus determinantes ha adoptado enfoques más proactivos y preventivos en consonancia con principios, entre ellos la defensa de la equidad, enfatizados por la Organización Mundial de la Salud (OMS)^1^^–^^4^.

Las interpretaciones del concepto de equidad son amplias^5^^,^^6^ y comprenden desde el alineamiento al antiintervencionismo estatal a la defensa de la igualdad de capacidades básicas y el máximo respaldo del Estado, pasando por la defensa de la equidad en el acceso^6^. Las primeras posturas se distancian de los principales debates sobre la equidad al asentarse en la premisa de que la desigualdad es inherente a la libertad de elección, si bien algunos autores optan por fijar un conjunto de servicios básicos^7^^,^^8^. Las demás alternativas, en mayor o menor medida defienden el diseño de políticas con la finalidad de atenuar o erradicar las inequidades en salud. Entre estas últimas, los estados tienen un papel más relevante^9^, con propuestas orientadas principalmente a defender la universalidad –cuyo referente es el *Beveridge Report*^10^–, a proveer sistemas de seguridad social y a introducir los llamados *means-testing programs*^11^^,^^12^ –una suerte de rentas de inserción social o discriminación positiva–, formas, todas ellas, de intervención no exentas de debate^13^^–^^15^.

La OMS^(^^1^^–^^4^^)^ se aproximó al concepto de “capacidades básicas” con base en las aportaciones de Sen^16^^,^^17^ y Nussbaum^18^^,^^19^, que acepta las responsabilidades personales en el estado de salud y defiende la actuación sobre cualquier factor causante de inequidad –condiciones socioeconómicas, sociodemográficas, geográficas, étnicas o de género desfavorables– que pueda introducir desigualdades de bienestar. De la propuesta deriva el concepto “desigualdad evitable”^5^ desarrollado por la OMS.

España, al igual que los otros países europeos y de la Organización para la Cooperación y el Desarrollo Económicos (OCDE), ha incluido el principio de equidad en las políticas de salud, acentuando su asimilación durante la primera década de este siglo y revisando su pervivencia en el contexto de la crisis financiera de 2008^20^^–^^23^.

Dejando al margen el tratamiento de la pertinencia de las diferentes aplicaciones de la equidad a la realidad social, este trabajo se interesa por el modo en que los servicios sanitarios de las Comunidades Autónomasᵃ (en adelante, CC.AA.) introducen la cuestión de la equidad y qué colectivos o circunstancias tienen en cuenta al abordarla. El objetivo es revelar la presencia del concepto de equidad en los planes de salud, identificar en ellos los principales temas asociados y el tratamiento que se les concede, contrastando sus orientaciones en el marco de las políticas públicas de salud con las visiones auspiciadas por el Ministerio de Sanidad, la Unión Europea (UE) y la OMS.

## MÉTODOS

### Diseño

Siguiendo las pautas del análisis comparativo constante^24^, mediante el examen de contenidos^25^^–^^27^ apoyado en una aplicación de soporte a la investigación cualitativa –Nvivo12–, se han revisado los planes de salud de las diferentes CC.AA.

### Muestra

El estudio comprende los planes generales de salud vigentes –de nueva elaboración o prorrogados– promovidos por 16 CC.AA.

- Andalucía: IV Plan Andaluz de Salud (2013–2020)
- Aragón: Plan de Salud Aragón 2030
- Baleares: Plan Estratégico 2016–2020
- Canarias: Plan de Salud de Canarias 2016–2017: Entre la crisis y la transformación para la innovación en la gestión de la salud de los servicios
- Cantabria: Plan de Salud de Cantabria 2014–2019
- Castilla-La Mancha: Estrategia 20-20 de Salud y Bienestar Social: El modelo de Castilla-La Mancha
- Castilla y León: IV Plan de Salud de Castilla y León (2020)
- Cataluña: Plan de salud de Cataluña 2016–2020
- Comunidad Valenciana: IV Plan de Salud 2016–2020 Comunitat Valenciana
- Extremadura: Plan de Salud de Extremadura 2013–2020
- Galicia: Estrategia Sergas 2020
- La Rioja: III Plan de Salud de La Rioja (2013–2020)
- Navarra: Plan de Salud de Navarra 2014–2020
- País Vasco: Plan de Salud Euskadi 2013–2020
- Principado de Asturias: Plan Sociosanitario del Principado de Asturias (2018–2021)
- Región de Murcia: Plan de Salud de 2010–2015 de la Región de Murcia

Dichos planes forman parte del estudio al cumplir los criterios de homogeneidad, representatividad, pertinencia y exhaustividad que propone Bardin^25^. Se buscaba analizar los planes generales sobre salud lo más actuales posibles. Es por ello que no se ha podido analizar ningún plan de la autonomía de Madrid dado que siguiendo la regla de la homogeneidad no se pudo encontrar un plan con estas características, siendo todos los recabados parciales y específicos.

### Análisis

La literatura identifica factores o determinantes de la salud, que, por influencia de las instituciones transnacionales de referencia, era esperable encontrar reproducidos, en mayor o menor medida, en los planes de salud.

Sin embargo, el análisis de contenido desde la perspectiva de Bardin^25^, especialmente del enfoque emergente^24^ adoptado, obliga a extraer del objeto de estudio las categorizaciones y significados, y no a prefijarlos.

Así, durante la fase de preanálisis^25^, se procedió a la selección de los fragmentos de los planes en que se abordaban contenidos vinculados a la equidad. Desde esta primera aproximación, se detectaron varios ítems claves y se expandió la selección de fragmentos de texto –en consecuencia con la perspectiva emergente adoptada– a otros dos campos de significados que se sintetizan en los conceptos de “Accesibilidad” e “Igualdad”, que se han denominado nodos o categorías base (en adelante, CB). El concepto “nodo” es propio de la terminología de la aplicación Nvivo y se refiere a una categoría que agrupa significantes similares: cada categoría opera como conjunto de semas relacionados con aquel que la nombra^b^.

Posteriormente se procedió al análisis de contenido y, como es propio de un enfoque de comparación constante, se conformaron nuevas subcategorías^c^ según la frecuencia de la aparición. Algunas de ellas ampliamente recogidas en la bibliografía consultada, y otras, menos comunes, derivadas del diseño adoptado. Como resultado, se fijaron las siguientes categorías clave (en adelante, CC): Calidad, Diversidad Funcional, Género, Grupos Sociales Vulnerables, Inmigración, Personas Mayores, Rural y Enfermedades Mentales.

La diferencia entre las CB y CC radica en su función en el proceso de análisis: las “base” articulan la selección de los segmentos del plan analizados (operan como significantes marco); y las “clave” determinan el contenido dentro de esa selección. En otras palabras, las categorías base son los contenedores (los segmentos de texto englobados en el análisis) de lo que se habla o temas tratados (categorías clave). Este es otro de los motivos que ha llevado al equipo investigador a aceptar la “multicodificación” en estos tres casos, dado que permite realizar un análisis temático de las CC ajustado a cada una de las CB y, a partir del mismo, establecer las similitudes y diferencias entre los distintos paradigmas representados.

En el Cuadro 1 se recogen las operacionalizaciones realizadas para conformar las diversas categorías base y clave que se han mencionado en el artículo, así como los términos o lexemas que se incluyeron en el análisis^d^.

**Cuadro 1:** Relación de categorías y de los términos o lexemas aplicados y englobados en la codificación.

Categoría		Términos o lexemas empleados
Categorías Base	Equidad	Equidad, Equitativo*, Inequitativ*
	Igualdad	Igualdad, Desigualdad*, Igualitar*
	Accesibilidad	Accesibilidad, Accesible, Acceso, Inaccesible, Inaccesibilidad
Categorías Clave	Diversidad funcional	Diversidad funcional, Discapacitad*, Discapacidad, Dependient*, Dependencia, Independencia
	Rural	Rural, Periferia, Núcleos poblacionales pequeños/alejados, Dispersos, Dispersión, Difícil acceso, Alejad*, Zona*, Geografía*, Geográfic*
	Grupos sociales vulnerables	Grupo social, Grupos sociales, Colectivos en situación de riesgo, Vulnerabilidad, Vulnerabl*, Desfavorecid*, Colectivo*
	Género	Género, Hombre*, Mujer*, Violencia, Maltrato, Machismo
	Inmigración	Inmigración, Inmigrante, Raza, Etnia, Étnic*, Lengua, Habla, Extranjer*
	Personas Mayores	Mayor*, Tercera Edad/3ᵃ Edad, envejecer, envejecimiento, ancian*
	Calidad	Calidad
	Mental	Mental*

* Se han seleccionado aquellas palabras que empiezan de ese modo, de forma que se pueden usar para obviar el género o los plurales de las mismas. Se trata de una práctica propia del programa de Nvivo y de sus criterios de búsqueda, por ello se plasma de esa manera. Estos términos se han revisado caso a caso para observar la pertinencia de los mismos, descartando los que no tenían un significado coherente con lo buscado.

## RESULTADOS

En primer lugar, se ha determinado en qué medida los planes de salud contienen referencias a los conceptos asociados a la equidad en salud (Figura 1).

**Figura 1:** Porcentaje de texto codificado según las categorías base sobre el total del texto recogido en los planes autonómicos.

Tal y como se aprecia, el concepto más extensamente detectado dentro de los planes de salud es el de Accesibilidad, con un 2,06% (de media), superior al tratamiento de las restantes CB en 11 de los planes autonómicos de salud analizados. Sin embargo, el concepto Igualdad alcanza mayores cotas de mención (en cuanto a máximos, siendo su media inferior con un 1,87%), destacando en el 6,53% de los contenidos del plan de salud de Andalucía, el 6,12% del plan del País Vasco y el 4,51% en el caso de Aragón.

A su vez, el concepto Equidad tiene una mayor relevancia en el plan de salud del País Vasco (4,23% de contenidos) y, en menor medida, en el plan autonómico andaluz (1,77%) y en el de las Islas Canarias (1,67%), obteniendo una media de codificación del 1,08%, la menor de las tres CB.

Por su parte, el concepto Accesibilidad alcanza su presencia más elevada en el plan autonómico de Castilla-La Mancha (3,92%), seguido por el del País Vasco (3,33%) y el de Asturias (2,84%).

Resulta, asimismo, llamativa la ausencia de contenidos hallada en algunos planes: el tratamiento del concepto Igualdad es exiguo –menos del 0,25%– en los planes de Asturias, Islas Baleares y La Rioja; el concepto Equidad es mínimamente abordado –menos del 0,7%– en los planes de Islas Baleares, Cantabria y Castilla-La Mancha; y el término Accesibilidad alcanza sus cifras más bajas –menos del 1,2%– en los planes de Islas Baleares, Murcia y Extremadura.

Pero ¿a qué se refiere cuando se habla de equidad, igualdad y accesibilidad?

Aunque, como se señaló, los temas asociados a las CB –Equidad, Igualdad y Accesibilidad– son incorporados a la práctica en totalidad de los planes de salud, el tratamiento de CC encuentra diferencias significativas, según muestra la distribución de menciones en los planes de salud autonómicos (Tabla 1).

**Tabla 1:** Menciones a las categorías base y claves en los planes de salud autonómicos por frecuencia de codificación (sobre total codificado por categoría).

	Accesibilidad	Calidad	Diversidad funcional	Equidad	Género	Grupos Sociales Vulnerables	Igualdad	Inmigración	Personas Mayores	Enfermedades Mentales	Rural	Media de menciones sobre total por CC. AA.
Andalucía	5,38%	10,86%	4,34%	8,01%	7,61%	5%	16,90%	5,36%	0,67%	6,52%	10,10%	7,34%
Aragón	3,27%	1,33%	0,91%	4,14%	0,74%	3,50%	7,99%	5,85%	4,67%	2,10%	8,75%	3,93%
Baleares	2,16%	0,27%	0,16%	0,54%	0%	0%	0,17%	1,17%	0%	0%	0%	0,41%
Canarias	6,13%	6,63%	0,99%	10,14%	0,34%	3,88%	5,24%	1,17%	0%	2,05%	3,52%	3,64%
Cantabria	16,56%	3,71%	18,85%	11,04%	3,69%	8,13%	5,52%	10,62%	5,71%	52,70%	5,44%	12,91%
Castilla-La Mancha	2,78%	0,80%	3,28%	0,81%	0,11%	0,48%	1,25%	0,39%	0,67%	0%	3,47%	1,28%
Castilla y León	6,93%	2,12%	7,18%	7,51%	2,61%	1,67%	7,10%	4,42%	0,46%	6,52%	9,74%	5,11%
Cataluña	5,99%	42,71%	1,53%	3,68%	58,55%	19,82%	2,65%	0%	59,18%	2,09%	0%	17,84%
Comunidad Valenciana	8,41%	3,98%	4,13%	12,43%	3,98%	21,28%	17,63%	16,17%	0,64%	0%	0%	8,06%
Extremadura	10,98%	7,43%	13,31%	11,92%	10,34%	5,88%	10,23%	9,97%	17,08%	2,17%	47,28%	13,33%
Galicia	1,61%	1,06%	0%	1,17%	0%	0%	0,18%	0%	0%	0%	0%	0,37%
La Rioja	10,97%	5,04%	22,17%	6,91%	6,59%	7,55%	2,55%	30,04%	6,26%	8,70%	6,01%	10,25%
Navarra	6,42%	3,45%	9,32%	3,86%	0,68%	7,27%	6,08%	7,60%	0,96%	2,11%	3,11%	4,62%
País Vasco	6,39%	8,22%	1,94%	14,58%	4,03%	7,65%	13,86%	0,81%	2,03%	1,98%	0,62%	5,65%
Principado de Asturias	3,02%	1,59%	6,86%	0%	0,45%	4,37%	0,91%	0,39%	1,67%	10,87%	1,29%	2,86%
Región de Murcia	3%	0,80%	5,03%	3,26%	0,28%	3,52%	1,74%	6,04%	0%	2,19%	0,67%	2,41%
Total Menciones a la categoría	100,00%	100,00%	100,00%	100,00%	100,00%	100,00%	100,00%	100,00%	100,00%	100,00%	100,00%	100,00%

Nota: Los colores atienden a la intensidad y dirección de la codificación; aquellos más próximos al rojo denotan menor porcentaje de mención; los verdes mayor; y los tonos amarillos los más moderados. En este caso representa los valores en columna y responde, por lo tanto, al porcentaje de segmentos del texto que se han codificado en cada categoría según el total individual de cada una de ellas. Como resultado, se muestra qué comunidades enfatizan en mayor o menor medida cada tema.

En las columnas que se hace mención al número de páginas de los planes, se crea un indicador en la última columna que atiende al ratio de codificación media por página del plan, que permite relacionar el porcentaje de codificación de cada comunidad con su número de páginas: cuanto más verde mayor es el ratio entre páginas y codificación y, por ende, mayor la atención prestada a la equidad; y los más próximos al rojo figuran las comunidades que menor atención prestan.

Desde el punto de vista de la diversidad temática abarcada, la menor variedad de CC se detecta en los planes de Galicia y Baleares –en Galicia solo se trata el concepto Calidad y en Islas Baleares, Inmigración y Calidad– mientras que son varios los planes autonómicos de salud (Andalucía, Aragón, Cantabria, Castilla y León, Extremadura, La Rioja y Navarra) que comprenden la totalidad de CC, si bien con desigual atención.

A su vez, respecto a la intensidad de tratamiento temático, destaca el plan de Cataluña, que concentra más de la mitad de las menciones que a tres CC se hacen en los planes autonómicos: Personas Mayores (59,18%), Género (58,55%) y Calidad (42,71%). Otros dos planes autonómicos reúnen la mayor parte de referencias a dos CC: Enfermedades Mentales (52,7%), en el caso de Cantabria, y Rural (47,28%), en de Extremadura.

Con menor intensidad, otras CC en las que despuntan los planes de salud son: Inmigración (La Rioja, 30,04%), Grupos Sociales Vulnerables (Cataluña, 21,28%; Comunidad Valenciana, 19,82%) y Diversidad Funcional (La Rioja, 22,17%; Cantabria, 18,85%).

Por otra parte, es importante situar el tratamiento de los temas en el contexto de la relevancia que tienen en los planes, ya que no necesariamente el que un plan acumule el mayor número de menciones a una categoría implica que a esta se le conceda mayor importancia respecto a las demás, como se podrá ver a continuación.

En primer lugar, cabe destacar que las CB poseen mayor presencia, algo lógico dada la selección del texto en función de las mismas. En este sentido, la CB que más codificación acumula de media es Accesibilidad, con un 36,09% de las menciones; seguida por Igualdad, un 22,77%; y Equidad, 16,24%. Esta última mantiene un porcentaje de menciones similar al alcanzado por la CC Diversidad Funcional (13,72%), uno de los temas más importantes de los fragmentos seleccionados.

Las restantes CC se ven subsumidas en cuanto a número de menciones: Grupos Sociales Vulnerables (3,77%), Personas Mayores (2,42%), Inmigración (2,19%), Género (1,17%), Rural (1,31%), Calidad (0,30%) y Enfermedades Mentales (0,03%).

Ponderando el peso de las CC al margen de las CB se facilita un análisis temático más afinado por comunidad (Tabla 2). Así, se observa que la categoría Diversidad Funcional destaca rotundamente sobre las demás (48,36% de media, con solo Aragón, Cataluña y Galicia por debajo del 30% de menciones) y hay que descender hasta el 16,56% de las codificaciones medias para encontrar la siguiente CC, Grupos Sociales Vulnerables – –destacan las menciones a este *issue* en los casos de la Comunidad Valenciana, País Vasco, Canarias, Cataluña y Aragón, todas por encima de un 20% de la codificación temática realizada mediante las CC–, o hasta el 11,40% para encontrar la tercera categoría, Inmigración, que destaca su peso para las Islas Baleares con un 55,37% y en menor medida Aragón y la Comunidad Valenciana con cifras en torno al 20%.

**Tabla 2:** Porcentaje de codificación de los *issues* abordados por las CC (sobre total codificado por planes de salud autonómicos).

	Calidad	Diversidad funcional	Género	Grupos Sociales Vulnerables	Inmigración	Personas Mayores	Enfermedades Mentales	Rural	Total
Andalucía	2,62%	47,76%	8,55%	16,54%	10,48%	1,45%	0,21%	12,41%	100,00%
Aragón	0,58%	18,08%	1,48%	20,98%	20,72%	18,53%	0,13%	19,50%	100,00%
Baleares	1,47%	43,16%	0,00%	0,00%	55,37%	0,00%	0,00%	0,00%	100,00%
Canarias	4,88%	33,77%	1,19%	39,58%	7,12%	0,00%	0,13%	13,32%	100,00%
Cantabria	0,30%	73,85%	1,48%	9,57%	7,41%	4,45%	0,54%	2,39%	100,00%
Castilla- La Mancha	0,43%	81,14%	0,30%	3,58%	1,71%	3,28%	0,00%	9,56%	100,00%
Castilla y León	0,48%	71,93%	2,69%	5,04%	7,87%	0,91%	0,19%	10,89%	100,00%
Cataluña	3,55%	5,83%	22,77%	22,77%	0,00%	45,07%	0,02%	0,00%	100,00%
Comunidad Valenciana	0,64%	29,42%	2,89%	45,61%	20,52%	0,92%	0,00%	0,00%	100,00%
Extremadura	0,60%	49,75%	3,94%	6,60%	6,63%	12,73%	0,03%	19,71%	100,00%
Galicia	100,00%	0,00%	0,00%	0,00%	0,00%	0,00%	0,00%	0,00%	100,00%
La Rioja	0,34%	68,18%	2,07%	7,00%	16,45%	3,83%	0,06%	2,07%	100,00%
Navarra	0,55%	68,81%	0,52%	16,15%	9,99%	1,39%	0,03%	2,56%	100,00%
País Vasco	3,26%	35,43%	7,58%	42,19%	2,68%	7,46%	0,12%	1,28%	100,00%
Principado de Asturias	0,39%	77,62%	0,53%	14,93%	0,78%	3,79%	0,33%	1,63%	100,00%
Región de Murcia	0,23%	69,01%	0,41%	14,51%	14,74%	0,00%	0,09%	1,02%	100,00%
Media de mención a las CB por CC. AA.	7,52%	48,36%	3,52%	16,56%	11,40%	6,49%	0,12%	6,02%	100,00%

Nota: Para una mayor claridad del peso de los contenidos dentro de cada plan, se pormenoriza en la frecuencia de mención relativa de cada una de las CC y se analiza el peso específico de cada una de ellas sobre las restantes (excluyendo, como se ha comentado, las CB). Así se destacan aquellos temas o *issues* abordados en mayor o menor medida por cada comunidad. El uso de colores sigue el patrón anteriormente aplicado, pero en este caso responde a las cifras ubicadas en las filas, por lo que muestra la importancia relativa de la CC en cada uno de los planes de forma individualizada y, por ende, en cada comunidad.

La supremacía del concepto Diversidad Funcional está presente en la mayoría de las CC. AA., siendo uno de los *targets* más destacados –resulta especialmente visible en los planes de Castilla-La Mancha, Asturias, Cantabria, Castilla y León, Región de Murcia, La Rioja y Navarra–. Queda al margen Galicia, que aborda de manera exclusiva la equidad en salud relacionándola con la Calidad.

La centralidad de la Diversidad Funcional asociada a la categoría Accesibilidad puede explicarse a partir de las medidas expuestas en los planes de salud y en cómo se argumentan sus causas: desde el acceso a las propias infraestructuras sanitarias hasta la adaptación de las páginas web a formatos más accesibles para los diferentes tipos de usuarios, pasando por el establecimiento de atenciones domiciliarias que faciliten la prestación de la atención sanitaria en condiciones de igualdad.

Las categorías Diversidad Funcional, Grupos Sociales Vulnerables e Inmigración centran la atención en la mayoría de contenidos que expresan la necesidad de potenciar la equidad en salud, articulando políticas o medidas que procuran mejorar la situación en que se encuentran estos colectivos.

En ese sentido, teniendo en cuenta que los campos semánticos de cada categoría implican no solo la consideración de una condición o unas circunstancias, sino que aluden a la porción de población por estas afectada, cabe destacar que entre los planes examinados también resultan principales las acciones en salud encaminadas a mejorar la situación de otros colectivos: Cataluña respecto a la CC Personas Mayores (45,07%) y Género (22,77%); o Extremadura y Aragón, al tratar la CC Rural (19,71% y 19,50%, respectivamente).

A partir de todos los datos anteriores, se ha representado (Figura 2) la situación de cada plan de salud según la relevancia concedida a cada CC.

**Figura 2:** Ranking de las CCAA según la atención que prestan a las distintas CC en sus planes.

A partir de esta figura se observa que hay varias CC. AA. cuya aproximación a la equidad se concentra en pocas CC, mientras otras abordan más *targets* y, por tanto, entienden que es una cuestión más amplia. En el primer grupo figuran Baleares, La Rioja, Navarra, Región de Murcia, Castilla y León, Cantabria, Asturias, Castilla-La Mancha y, finalmente, Galicia; en el segundo, Aragón, Andalucía, Canarias, Cataluña, Extremadura, País Vasco y la Comunidad Valenciana, todas ellas por debajo de la media de desviación típica y, por tanto, que tienden a observar y tener en cuenta más colectivos que las restantes.

## DISCUSIÓN

Conocer cuál es la aproximación de cada comunidad a la equidad, en qué colectivos están centrando sus políticas sanitarias y con qué orientación, implica comprobar qué cuestiones no se están abordando, lo que plantea la pregunta sobre dicha ausencia teniendo en cuenta la consideración que actores transnacionales como la OMS y la propia UE poseen de la teoría de las capacidades básicas propuesta por Sen^3^^,^^4^^,^^16^^,^^17^^,^^21^.

Este estudio cualitativo corrobora los hallazgos de investigaciones precedentes, de que los planes de salud autonómicos contienen escasas referencias explícitas al concepto de equidad en salud^20^^–^^23^^,^^28^. Los planes focalizan su atención al abordaje de enfermedades concretas, la incorporación de tecnologías sanitarias y la reorganización de servicios, dejando en un segundo plano la cuestión de la equidad, que solo se revela, indirectamente, por medio de la demanda de una mejora del acceso a los servicios sanitarios, adoptando, en suma, una visión orientada a la perspectiva del acceso en igualdad de condiciones y no acorde con la propuesta de Sen y la OMS sobre la igualdad de capacidades y de la atención a los determinantes sociales.

Además, este acceso no se considera por todas las CC.AA. como un factor general y multicausal con diversos colectivos o *issues* clave a tener en cuenta, sino que se significan colectivos concretos, entre los que destaca el de las personas con Diversidad Funcional, principal *target* al que se dirigen las políticas de equidad en salud. En un segundo plano quedan las referencias a otros colectivos destacados como los Grupos Sociales Vulnerables y Personas Inmigrantes. Lo anterior no impide resaltar que el análisis confirma que los planes de salud vinculan la categoría Grupos Sociales Vulnerables a situaciones de discriminación económica, relación puesta en evidencia por organismos transnacionales como la OMS como fuente de inequidad asociada a pobreza y empeoramiento de la salud^5^.

Por su parte, la atención a temas como género, calidad, personas mayores, ámbito rural o enfermedades mentales no se contempla de forma uniforme en los planes autonómicos. Sin embargo, este hecho no los convierte en cuestiones irrelevantes, al contrario, su ausencia evidencia la carente perspectiva multidimensional de las inequidades que existen en materia de salud. Todo esto tiene una grave repercusión, ya que puede comportar un déficit de atención a dichos colectivos, que padecen dificultades añadidas para poder ser usuarios de pleno derecho del servicio sanitario^20^^–^^23^^,^^28^^,^^29^.

Desde el punto de vista del promotor de los planes de salud –las Administraciones Autonómicas– se pueden distinguir, por una parte, comunidades que abordan la equidad como una cuestión muy focalizada en uno o dos colectivos y las que tratan el problema de una manera más comprehensiva, más global y próxima al concepto de capacidades básicas, si bien ninguna de ellas va más allá del factor accesibilidad y no tienen en cuenta completamente los demás componentes de la inequidad en salud como defienden la OMS y la literatura sobre el caso español^20^^–^^23^^,^^28^^–^^30^.

Aunque la concepción del diseño de las políticas de salud pueda adolecer de insuficiente atención específica a determinados colectivos, la implementación los debería tener en cuenta y evitar, así, graves inequidades en los niveles de uso del servicio: el acceso a un mismo servicio no garantiza una igual atención si, por ejemplo, algunos usuarios carecen de recursos económicos para sufragar su medicación, tienen dificultades para entender el idioma en que se les atiende o son incapaces de visualizar una prescripción. La calidad de su atención, incluso la misma atención, está en riesgo^6^^,^^16^^,^^17^^,^^19^.

Todas las categorías clave que se han detectado son perfectamente aplicables al conjunto de las CC. AA. No resaltarlas no deriva de la inexistencia de colectivos concretos, sino de una elección política de los *target* de los planes.

Confirmando lo establecido en investigaciones previas que apuntaban en la misma dirección^20^^–^^23^^,^^28^^–^^30^, los resultados certifican la falta de una mayor plasmación de la equidad en los planes y de en general abordar pocos de los temas clave. Depender de que solo la implementación cubra las carencias de la planificación y, como sucedería en este caso, pueda mitigar un exiguo tratamiento de la equidad, no resulta una práctica solvente.

Este último, el de la implementación, es un terreno situado más allá de los propósitos de este trabajo, y será en el estudio de la implementación de lo que los planes de salud proponen en que realmente se pueda constatar cómo y en qué se traducen las ideas de partida^20^^,^^31^ que han sido objeto de este análisis. En este sentido, cabrá esperar que esta investigación estimule nuevos estudios al respecto y sirva como marco que permita análisis más profundos de los planes de salud vigentes y venideros.

## Footnotes

a Cabe recordar que, según la vigente Constitución de 1978, España es un Estado descentralizado territorialmente en 17 comunidades con amplias competencias sobre diversas materias y servicios, entre ellos, los de salud. La Administración central se reserva las competencias sobre sanidad exterior y la coordinación del sistema sanitario en general, transfiriéndose el resto de competencias sobre los servicios de salud a las CC.AA. Dado el actual alto nivel de descentralización territorial, se podría afirmar que no hay un único sistema sanitario en el país, sino 17 subsistemas, pudiendo algunas comunidades ofrecer servicios diferentes, si bien en los aspectos básicos el sistema es relativamente homogéneo para el conjunto del Estado.

b Cabe reseñar que estas tres categorías contradicen el criterio de “exclusión mutua”, básico para la configuración de categorías, algo que el propio equipo de investigación pretendió evitar. Esta circunstancia forma parte de las excepciones que Bardin^25^ contempla para el fenómeno que denomina “multicodificación”. Es decir, dada la imprecisión con que en ocasiones se hace referencia a ideas o ítems conceptualizados de forma diferente por la literatura, se pueden codificar estos elementos sin que necesariamente su codificación dentro de una categoría los excluya de su pertenencia a otra. Esto ocurre con las denominadas CB, que son utilizadas en algunas ocasiones como sinónimos. Con la pretensión de lograr establecer si existen cuestiones específicas de cada una de ellas y, a su vez, encontrar aquellas otras que sean transversales a las tres, se ha optado en este caso por emplear esta opción, de forma que en los casos en que existan segmentos de texto que hagan una mención de 2 o incluso de 3 CB, se considere que los temas abordados sean parte de las tres categorías.

c Estas subcategorías responden a la configuración habitual de las categorías, siendo completamente excluyentes y encontrándose contenidas en alguna o todas las anteriores.

d Fruto del propio análisis de la frecuencia de los lexemas del contenido de los planes y gracias a la herramienta del programa Nvivo que permite dicho criterio de búsqueda por lexema, amparados así mismo por la ampliación a través de los principales diccionarios de sinónimos y la competencia lingüística nativa del equipo investigador.
